# Quercetin exhibits potent antioxidant activity, restores motor and non-motor deficits induced by rotenone toxicity

**DOI:** 10.1371/journal.pone.0258928

**Published:** 2021-11-12

**Authors:** Syeda Madiha, Zehra Batool, Saiqa Tabassum, Laraib Liaquat, Sadia Sadir, Sidrah Shahzad, Fizza Naqvi, Sadia Saleem, Sarwat Yousuf, Amber Nawaz, Saara Ahmad, Irfan Sajid, Asia Afzal, Saida Haider

**Affiliations:** 1 Neurochemistry and Biochemical Neuropharmacology Research Unit, Department of Biochemistry, University of Karachi, Karachi, Pakistan; 2 Dr. Panjwani Center for Molecular Medicine and Drug Research, International Center for Chemical and Biological Sciences, University of Karachi, Karachi, Pakistan; 3 Faculty of Life Science, Department of Biosciences, Shaheed Zulfiqar Ali, Bhutto Institute of Science and Technology (Szabist), Karachi, Pakistan; 4 Multidisciplinary Research Lab, Bahria University Medical and Dental College, Bahria University, Karachi, Pakistan; 5 Pakistan Navy Medical Training School and College, PNS Shifa, Karachi, Pakistan; 6 Department of Biomedical Engineering, Sir Syed University of Engineering and Technology, Karachi, Pakistan; 7 Department of Biological and Biomedical Sciences, Aga Khan University, Karachi, Pakistan; 8 Department of Biochemistry, Federal Urdu University of Arts, Sciences & Technology, Karachi, Pakistan; Florey Institute of Neuroscience and Mental Health, The University of Melbourne, AUSTRALIA

## Abstract

The rotenone-induced animal model of Parkinson’s disease (PD) has been used to investigate the pathogenesis of PD. Oxidative stress is one of the main contributors of neurodegeneration in PD. Flavonoids have the potential to modulate neuronal function and combat various neurodegenerative diseases. The pre- and post-supplementation of quercetin (50 mg/kg, p.o) was done in rats injected with rotenone (1.5 mg/kg, s.c). After the treatment, behavioral activities were monitored for motor activity, depression-like behavior, and cognitive changes. Rats were decapitated after behavioral analysis and the brain samples were dissected out for neurochemical and biochemical estimation. Results showed that supplementation of quercetin significantly (*p*<0.01) restored rotenone-induced motor and non-motor deficits (depression and cognitive impairments), enhanced antioxidant enzyme activities (*p*<0.01), and attenuated neurotransmitter alterations (*p*<0.01). It is suggested that quercetin supplementation improves neurotransmitter levels by mitigating oxidative stress via increasing antioxidant enzyme activity and hence improves motor activity, cognitive functions, and reduces depressive behavior. The results of the present study showed that quercetin pre-supplementation produced more significant results as compared to post-supplementation. These findings show that quercetin can be a potential therapeutic agent to reduce the risk and progression of PD.

## Introduction

Worldwide a large range of chemicals is used to control a variety of harmful organisms from soil, water, and plants. Pesticides (insecticides, herbicides, fungicides) are widely used in agricultural fields to eradicate different kinds of pests. However, the adverse effects of these pesticides on human and animal health are also well documented. Humans can be exposed to these pesticides directly from agricultural fields, household use, and indirectly through the diet [1]. Among the various kinds of pesticides, rotenone is a commonly used non-specific pesticide. Rotenone is derived from plant species of the genus *Derris* and *Lonchocarpus* [2]. It was primarily used as a fish poison, since then various studies have suggested that rotenone administration in animals induced Parkinson’s disease (PD)-like symptoms. For this reason, it has been used to develop an animal model of PD [3,4].

PD is considered the second most prevalent neurological disorder [5]. It is defined by the presence of cardinal motor symptoms such as tremor, bradykinesia, postural instability, and rigidity [5]. All these motor deficits are frequently predated by non-motor deficits such as sleep disorder, olfactory deficits, cognitive impairments, and depression [6]. In recent years non-motor symptoms of PD such as depression and cognitive changes are considered as an important part of the disease [7]. Cognitive impairment might occur due to the deficits of neurotransmitters such as acetylcholine, dopamine, serotonin, and also due to genetic mutation implicated in PD [8]. The prevalence of depression in PD ranges from 40 to 50% resulting from psychological and neurobiological factors and alterations in the monoamine levels (dopamine, serotonin, norepinephrine) [9,10]. PD is characterized by the degeneration of dopaminergic neurons in the substantia nigra pars compacta and downstream loss of dopaminergic neuronal inputs to the striatum. This results in the depletion of DA, the most consistent pathological feature found in PD patients [8,11]. The exact cause of PD is yet to be known, but during the past 20 years, the advances in research technologies have determined that PD is not caused by a single factor but several factors such as mitochondrial dysfunction, protein misfolding and aggregation, and the loss of calcium homeostasis are involved in the pathogenesis of PD leading to the loss of dopaminergic neurons in substantia nigra [12]. Proper mitochondrial function is needed for neuronal survival. Previous studies have demonstrated that environmental toxin, genetic mutations, and age induce mitochondrial impairments [13–15]. Pezzoli et al. [16] reported that more than 80% population worldwide has been associated with PD due to exposure to pesticides and rural living. Rotenone, a mitochondrial poison, inhibits complex-I (the main part of electron transport chain) and results in neuronal death. Moreover, it is also reported that complex-I activity is diminished in substantia nigra resulting in mitochondrial damage in the brain of PD patients [14]. Rotenone administration in rodents produced neuropathological hallmark features of PD such as mitochondrial impairment, which is one of the core components that contribute to the neurodegeneration in PD [2]. Rotenone affects the motor and non-motor functions in rodents and hence can be used to test the therapeutic strategy against PD in rodents.

For the treatment of PD, surgery and drug therapies are recommended but till today there is no cure for PD and the available therapeutic strategies are not able to halt or slow down the disease progression. Some studies have shown that naturally occurring compounds can be the best therapeutic strategy for the prevention and progression of the disease [17,18]. Moreover, the daily intake of natural compounds strengthens neuronal function and reduces the risk of neurodegenerative diseases [19]. Tribal communities used phytochemicals derived from fruits and vegetables for the medication against different diseases [19]. In the field of alternative medicine, flavonoids have now become a topic of interest due to their beneficial effects on different diseases. In search of new therapeutic approaches, quercetin a natural flavonoid, strong antioxidant, and free radical scavenger has gained much interest in recent years. Quercetin (3,3’,4’,5,7-pentahydroxyflavone) a polyphenolic compound is ubiquitously found in common fruits and vegetables such as onions, broccoli, and apples [20]. Studies have reported that quercetin is one of the most potent antioxidants having the ability to scavenge free radicals and controlling many biological processes involving oxidative stress [21]. Various studies show the neuroprotective effect of quercetin against different neurodegenerative diseases and neurotoxic insults [22–24]. Quercetin acts as a direct antioxidant because of the presence of two pharmacophores that is catechol and OH group within the molecule [25,26]. Previously, quercetin has been shown to improve mitochondrial complex-I activity. It has also been shown to restore the activity of the electron transport chain and attenuate motor impairment in the rotenone-induced rat model of PD [27,28]. Quercetin is also reported to reverse memory impairment in the PD rat model induced by 6-OH dopamine by enhancing the oxidative defense system [20]. However, the association of these effects with regional biogenic amines particularly in the striatum of rotenone intoxicated rats is not reported earlier. Therefore, the current study is conducted to identify the effects of quercetin on rotenone-induced motor and non-motor deficits, and neurochemical alterations in the rat model of PD with relevance to antioxidant effects of quercetin.

## Materials and methods

### Animals

In this study, forty (40) male Wistar rats (150–200 g; 3–4 months) were used. Naïve rats were purchased from the DOW University of health sciences (OJHA campus), Karachi, Pakistan. The rats that were showing normal general activity in their home cages were included in the study. The general activity included grooming, rearing, and cage crossings by the rat. Rats were kept separately at controlled room (23±2°C) temperature with free access to standard rodent diet [29] and water with a natural 12:12 light/dark cycle. Animals were housed individually and were allowed to familiarize for 3 days to reduce suffering. The procedures were approved by and registered in institutional Advanced Studies and Research Board (ASRB/01834/Sc) and executed in line with National Institute of Health Guide for Care and Use of Laboratory Animals (Publication No. 85−23, revised 2011). Animals were handled with care and maximum efforts were made to reduce pain, suffering and distress. The general health and condition of all animals were monitored daily.

### Reagents and chemicals

Hydrogen peroxide stock (35%) solution, thiobarbituric acid, ferric chloride, trichloroacetic acid, nitro blue tetrazolium, and dithiobisnitrobenzoic acid were purchased from British Drug House (BDH, Dorset, UK). Rotenone, quercetin, hydroxylamine hydrochloride, acetylthiocholine, octyl sodium sulphate (OSS), and all other reagents were purchased from Sigma Chemical Co. (St. Louis, USA). Sunflower oil was purchased from the local market.

### Experimental protocol

**Fig 1:**
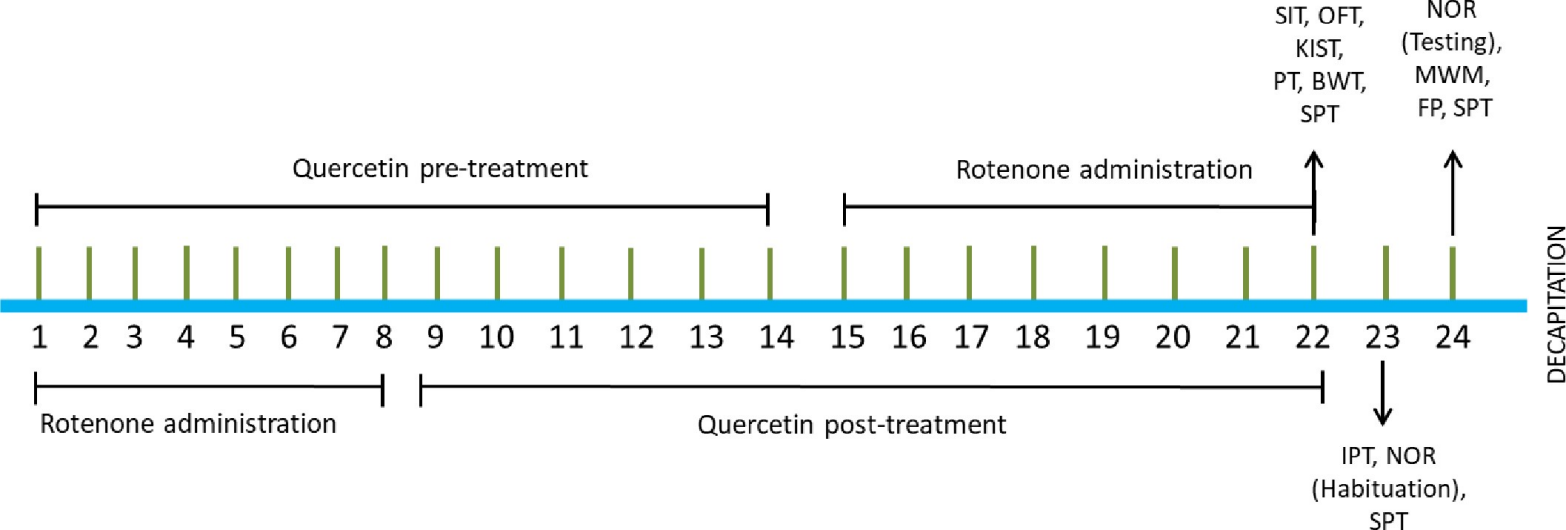
Schematic representation of experimental protocol. Behavioral analysis was monitored by using Kondziela inverted screen test (KIST), pole test (PT), beam walking test (BWT), open field test (OFT), inclined plane test (IPT), social interaction test (SIT), sucrose preference test (SPT), Morris water maze test (MWM), novel object recognition task (NOR), footprint (FP) test.

After three days of the acclimation period, rats (*n* = 40) were randomly divided into five groups (*n* = 8). Group I: Control, Group II: Quercetin (Que) (50 mg/kg, p.o.), Group III: Rotenone (Rot) (1.5 mg/kg, s.c), Group IV: Pre-Que+Rot and Group V: Post-Que+Rot. Rotenone was dissolved in slightly warm sunflower oil and was administered at the dose of 1.5 mg/kg [30]. Before starting the experiment, we analyzed our previous experimental data by power analysis in which we used rotenone and quercetin to identify the validity of sample size and we found the appropriateness of the sample size used in this study. We have reported neuroprotective effects of quercetin previously at 50 mg/kg [31,32], therefore, we selected the same dose in this experiment as well. The study was designed to determine the effects of pre- and post-treatment of quercetin in the rotenone-induced PD model. Group II and Group IV were administered with quercetin (dissolved in saline) at the dose of 50 mg/kg orally for two weeks (1^st^ to 14^th^ days). After two weeks of quercetin treatment, rotenone was administered subcutaneously at the dose of 1.5 mg/kg to Group IV for eight days (15^th^ to 22^nd^ days). In Group III and Group V, rotenone (1.5 mg/kg, s.c) was injected for 8 days to develop PD-like symptoms (1^st^ to 8^th^ days). After the development of PD-like symptoms quercetin (50 mg/kg, p.o) was administered in Post-Que+Rot group for two weeks (9^th^ to 22^nd^ days). The control group was treated with sunflower oil (s.c) and saline (p.o) as a vehicle (Table 1). Food intake and body weight were monitored daily throughout the treatment. Behavioral analyses were performed after completion of the respective treatment according to the schedule as shown in Fig 1. Motor activity was examined by Kondziela’s inverted screen test, pole test, beam walking test, open field test (OFT), inclined plane test, and footprint test. Non-motor behaviors including depression-like behavior and memory performance were also performed. Social interaction test and sucrose preference test were selected to examine depression-like behavior whereas novel object recognition task (NOR) and Morris water maze (MWM) were used to assess memory function. Behavioral tests were performed between 0800–1730 h. Rats were decapitated after 1 h of completion of behavioral analysis using guillotine without anesthesia. The decapitation was done using sharp blade of guillotine by a trained laboratory assistant to reduce suffering to the animals. Striatum and hippocampus were dissected out for neurochemical and biochemical estimations which were stored at -20°C. All experiments were conducted using a balanced design to avoid the effect of order and time of treatment and behavioral testing. The experimenters involved in the study were aware of the group allocation at the different stages of the experiment. There were no exclusions of animals, experimental units, or data points from the analysis.

**Table 1:** Drug dose and route of administration of groups.

Groups	N	Treatment
Control	8	Sunflower oil (s.c) + saline (p.o)
Quercetin	8	Sunflower oil (s.c) + quercetin (50 mg/kg) p.o
Rotenone	8	Rotenone (1.5 mg/kg) s.c + saline (p.o)
Pre-Que+Rot	8	Quercetin (50 mg/kg) p.o + rotenone (1.5 mg/kg) s.c
Post-Que+Rot	8	Rotenone (1.5 mg/kg) s.c + quercetin (50 mg/kg) p.o

Table 1. s.c–Subcutaneous, p.o–Per oral.

### Behavioral analysis

#### Kondziela’s inverted screen test

Kondziela test has been used previously to measure the muscular strength of animals using all four limbs [33]. The test was done by placing the rat in the center of the screen which was inverted for 120 s. The time when the rat fell off from the screen was noted.

#### Pole test

The pole test is used to assess basal ganglia-related movement disorders in rodents. In brief, rats were placed head-up on top of a vertical wooden pole (2.5 cm in diameter and 100 cm in height) and base of the pole was positioned in the home cage. The time taken by the rat to come down from the pole to the floor was noted [34].

### Beam walking test

Beam walking is a test of motor coordination. The rats have to cross a beam that is suspended between a start platform and their home cage at a height of 50 cm and is supported by two pillars. A cushion was placed under the beam to protect the animals from falling onto the floor. The difficulty of this task can be assorted by using beams with different widths [35]. Motor activity was assessed by the ability of a rat to cross different sizes of the beam. Three circular beams of different diameters were used in this study such as 3 cm, 2 cm, 1 cm, and length of 100 cm. In the training phase, animals were trained to traverse the three beams (from widest to narrowest). This helps to make certain that the behavior during testing is more stable and more precisely reflects motor coordination as opposed to the rodent’s natural aversion to crossing over unprotected spaces. After the training session testing phase was done and the time taken to cross the beams was recorded.

### Open field test (OFT)

The open field test is used to observe motor activity. The apparatus consists of a square (76 × 76 cm) with walls 42 cm high. Animals were placed individually in the central square of the open field. Latency to move in seconds and the number of squares crossed were monitored for five minutes [36].

### Inclined plane test

The cataleptic effect was investigated according to the previously described method by Costall and Naylor [37]. The test was done by gently placing the animal on an inclined surface for three minutes and the duration of time the animal remained in one position was recorded in seconds. Cataleptic score was calculated by (latency to move/total time) × 100) [38].

### Footprint test

To obtain the footprints, the rat hindlimb and forelimb were coated with green and red non-toxic paints. The animals were then allowed to walk along a 100 cm long, 10 cm wide runway (with 20 cm high walls). A fresh sheet of white paper was placed on the floor of the runway for each rat run. The footprint patterns were analyzed for three step parameters (all measured in centimeters): i) stride length, ii) base width, and iii) overlap between forelimb and hind limb. A sequence of four consecutive steps was chosen for evaluation, footprints made at the start and end of the run were not included [39].

### Social interaction test

The social interaction test is a simple test in which behaviors are video-recorded and analyzed to assess i) active interactions (sniffing, following, boxing, crawling under or over, aggressive behavior, social grooming, chasing), ii) passive interactions (close proximity with a distance of 5cm from skin to skin), and iii) number of interactions of a test rat with a novel rat [40,41]. The test was performed in a Perspex transparent activity box having dimensions of (26×26×26 cm) in a quiet room under bright white light. In each turn of testing, two rats were taken from two different groups for 5 minutes. Each rat was taken only once for testing per day.

### Sucrose preference test

This test is used as a measure of anhedonia in rodents [42]. This test was performed to assess animal interest in seeking out a sweetened drink over plain drinking water. Sucrose preference test was carried out in rat home cage. Rat cages were randomly allocated with two identical graduated water bottles for consecutive 3 days. One bottle contained plain drinking water while the second contained 250 ml of 1% w/v sucrose solution. Water and sucrose solution intake was measured daily and the position of two bottles was switched daily to reduce any confound produced by a location biasness. After testing, sucrose consumption was calculated by using the following equation % sucrose preference = sucrose intake×100/total intake.

### Novel object recognition task (NOR)

The memory function of rats was monitored by novel object recognition (NOR) task. The experimental apparatus used for the object recognition task was an open field box (40 × 40 × 40 cm) made of gray painted wood. The floor was covered with sawdust. The method was essentially the same as described earlier [43] with slight modification [44]. Memory function in NOR is assessed by monitoring the ability of rats to differentiate a novel object in a familiar environment [43,45]. To monitor memory function, rats were exposed to two similar objects A1 and A2 (transparent glasses filled with white cement) and a metallic container (novel object, B) filled with white cement. The test was divided into three phases, habituation, training, and testing phase. In habituation, the rat was exposed to the box for 10 min. On the second day, after 24 h of habituation, the training phase was started and the rat was exposed to two familiar objects inside the box for 5 min. After 5 min the rat was removed and placed back in the home cage. The test phase was carried out for 3 min after 20 min of the training phase [46]. During the test session, one familiar object was replaced by the new object (B). Sniffing time was recorded for exploring novel and familiar objects. The preference index was calculated as described earlier [47]. It is the ratio of exploration time for the new object and total exploration time for both familiar and novel objects.

### Morris water maze test (MWM)

Spatial and working memory function in rodents is determined through MWM [48]. In this test, rats find a hidden platform located underneath the water in a pool. The escape platform is a metallic cylinder having a smooth top surface with 8 cm diameter, placed 2 cm beneath the water surface. The platform is made invisible by the addition of milk in the water. In this test, MWM tank was divided into four quadrants namely NW, SW, SE, NE, and NW was selected as the target quadrant. During initial training for 2 min, each rat was introduced into the tank from each quadrant and allowed to locate the hidden platform. There was a gap of 15 min between each trial. Memory performance was assessed in a probe trial after 60 min of training, during which platform was removed and i) escape latency (s), ii) time spent in the target quadrant (s), and iii) crossing over target quadrant were recorded.

### Neurochemical analysis

#### Determination of dopaminergic and serotonergic levels

For the determination of biogenic amines, homogenization of frozen brain regions was carried out in an extraction medium using an electrical homogenizer (Polytron; Kinematica). The neurochemical analysis was done to assess concentrations of DA, DOPAC, 5-HT, and 5-HIAA in striatum and hippocampus. Reversed-phase High Performance Liquid Chromatography (HPLC) with an electrochemical detector (Schimadzu LEC 6A detector) was performed to detect levels of biogenic amines in brain samples. The EC detector was operated at a potential of +0.8 V. The stationary phase used for separation was a 5-μ Shim-pack ODS column having an internal diameter of 4.0 mm and a length of 150 mm. The mobile phase containing octyl sodium sulfate (0.023%) in 0.1 M phosphate buffer at pH 2.9 was passed through the column with the pump pressure of 2000–3000 psi [49].

### Determination of striatal acetylcholine (ACh) content and acetylcholinesterase (AChE)

The method as explained by Batool et al. [49] was used to estimate acetylcholine (ACh) content in the striatum. The tissue sample was boiled to inactivate the enzyme and release the bound ACh which reacts with ferric chloride and the brown color developed was read at 540 nm against the reagent blank. The concentration of ACh was expressed as μmol/g of tissue. The activity of AChE in the homogenate was determined according to the method of Haider et al. [50]. The activity of AChE was expressed as mmol/g/min of brain tissue.

### Biochemical analysis

For the estimation of oxidative status, a 10% (w/v) tissue homogenate was prepared with 0.1 M phosphate buffer (pH = 7.4) and centrifuged at 10,000×g for 10 min at 4°C. The supernatant was used for the estimation of lipid peroxidation [51], reduced glutathione (GSH) [52], superoxide dismutase (SOD) [53], glutathione peroxidase (GPx) [54], and catalase (CAT) [55] activities.

### Statistics

Data are presented as mean±SD. Mean differences were evaluated by one-way ANOVA followed by Tukey’s post-hoc test using SPSS version 20. Values *p*<0.05 were considered as significant.

## Results

### Food intake and body weight

**Fig 2:**
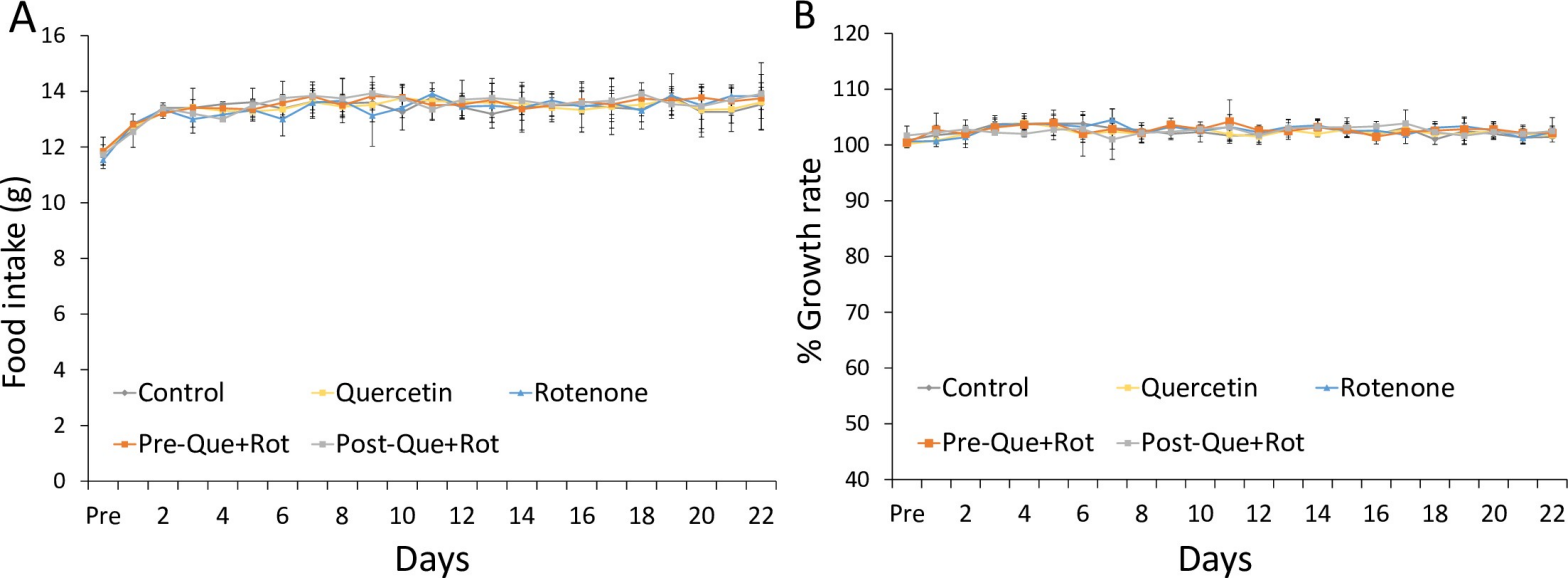
Effects of rotenone and pre-and post-administration of quercetin on food intake (A) and growth rate (B).Values are mean±SD (n = 8). Non-significant difference was obtained by one-way ANOVA following Tukey’s test. Growth rate in % was calculated by (Present weight/Initial weight) × 100).

The food intake and body weight measured to monitor the general health following the administration of rotenone were not affected among the groups (Fig 2) showing general health was not changed in this study.

### Quercetin supplementation improved motor symptoms of PD

**Fig 3:**
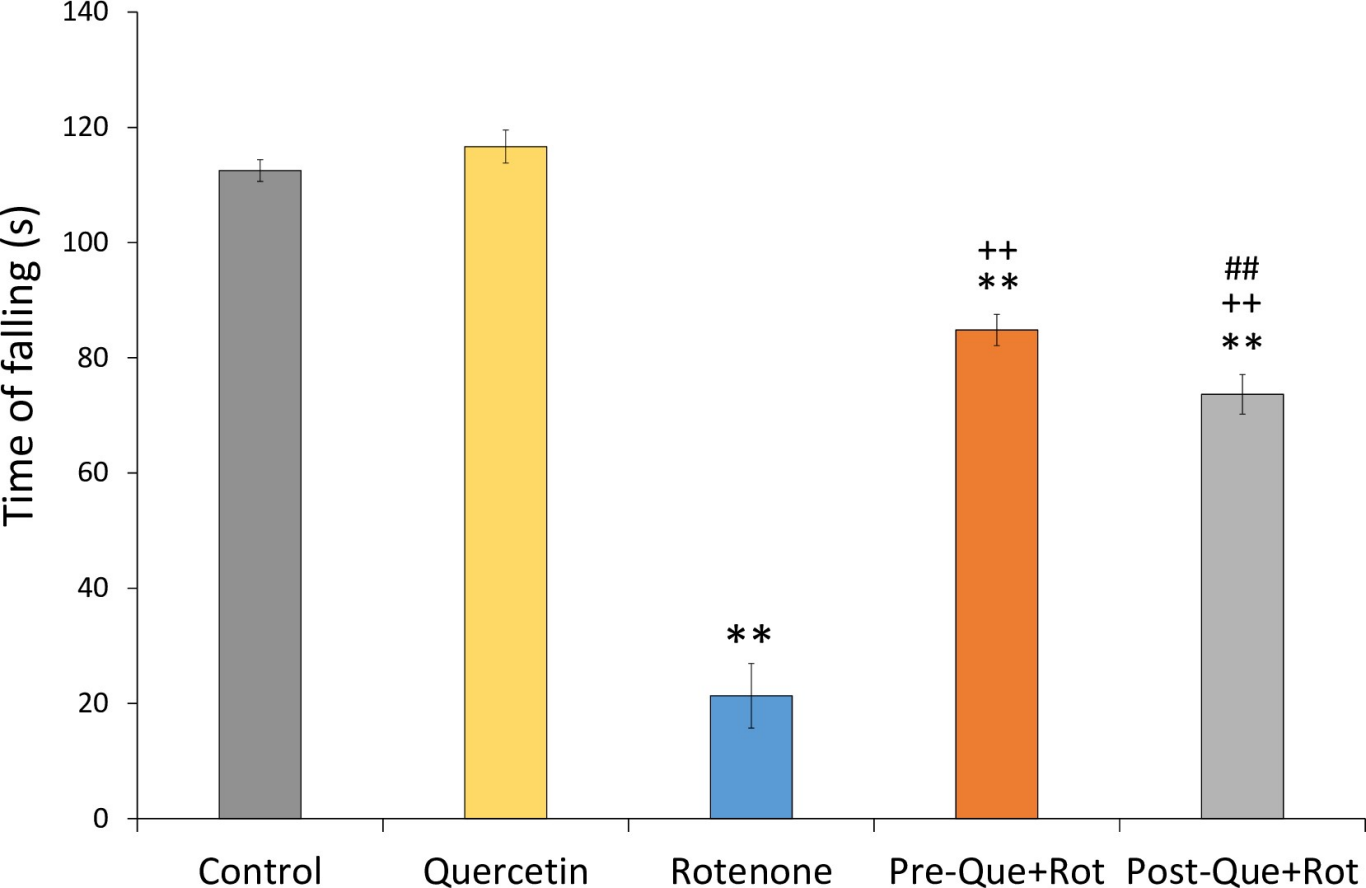
Effects of pre- and post-supplementation of quercetin on Kondziela inverted screen test in rotenone-injected rats. Significant differences were obtained by one-way ANOVA followed by Tukey’s test. **p<0.01 as compared to the control group; ++*p*<0.01 as compared to rotenone group; ##*p*<0.01 as compared to Pre-Que+Rot group.

Kondziela’s inverted screen test was used to monitor the muscular strength of rats (Fig 3). Data analyzed by one-way ANOVA showed a significant effect of treatment on muscular strength [*F* (4, 35) = 1320.039, *p*<0.01]. Post-hoc analysis revealed that the time of falling was significantly reduced in the rotenone group as compared to control animals (*p*<0.01). However, pre- and post-supplementation of quercetin significantly increased the time of falling (*p*<0.01) as compared to rotenone administered group. Furthermore, the results also demonstrated that supplementation of quercetin before the exposure of toxin substantially enhanced muscular strength (*p*<0.01) and improved the gripping ability as compared to that of post-supplemented group.

**Fig 4:**
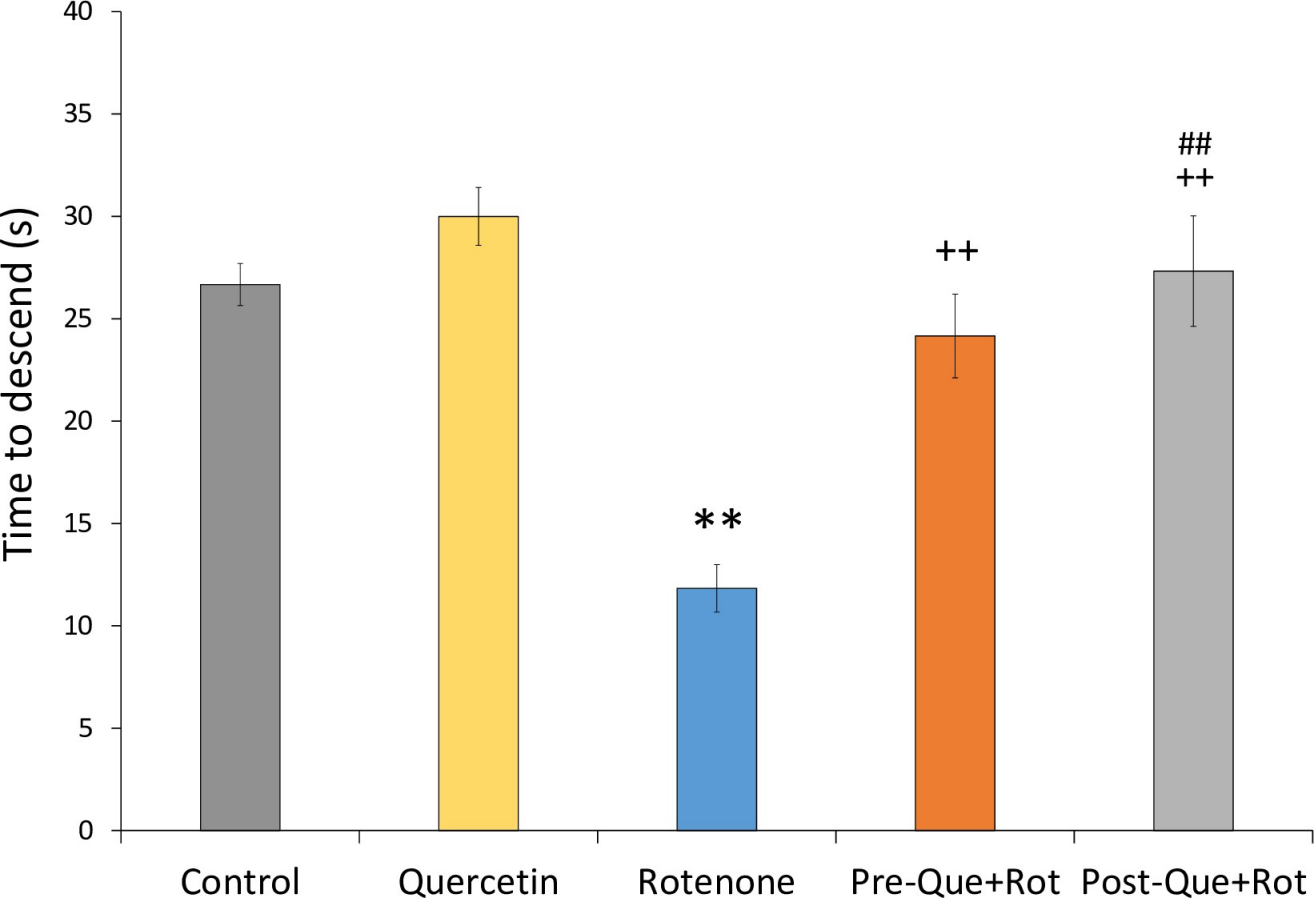
Effects of pre- and post-supplementation of quercetin on pole test in rotenone-injected rats. Significant differences were obtained by one-way ANOVA followed by Tukey’s test. **p<0.01 as compared to the control group; ++*p*<0.01 as compared to rotenone group; ##*p*<0.01 as compared to Pre-Que+Rot group. Values are mean±SD (*n = 8*).

Pole test was used to monitor the movement related to basal ganglia (Fig 4). Analysis of data by one-way ANOVA showed that treatment had a considerable impact on time to descend from the pole [*F* (4, 35) = 176.275, *p*<0.01]. In comparison to controls, the rats administered with rotenone took significantly less time to descend (*p*<0.01) from the pole while the rotenone groups treated with quercetin exhibited a significant increase in descending time (*p*<0.01) as compared to the group treated with rotenone alone. Moreover, in comparison to the post-treatment, pre-treatment with quercetin had more significant effects (*p*<0.01).

**Fig 5:**
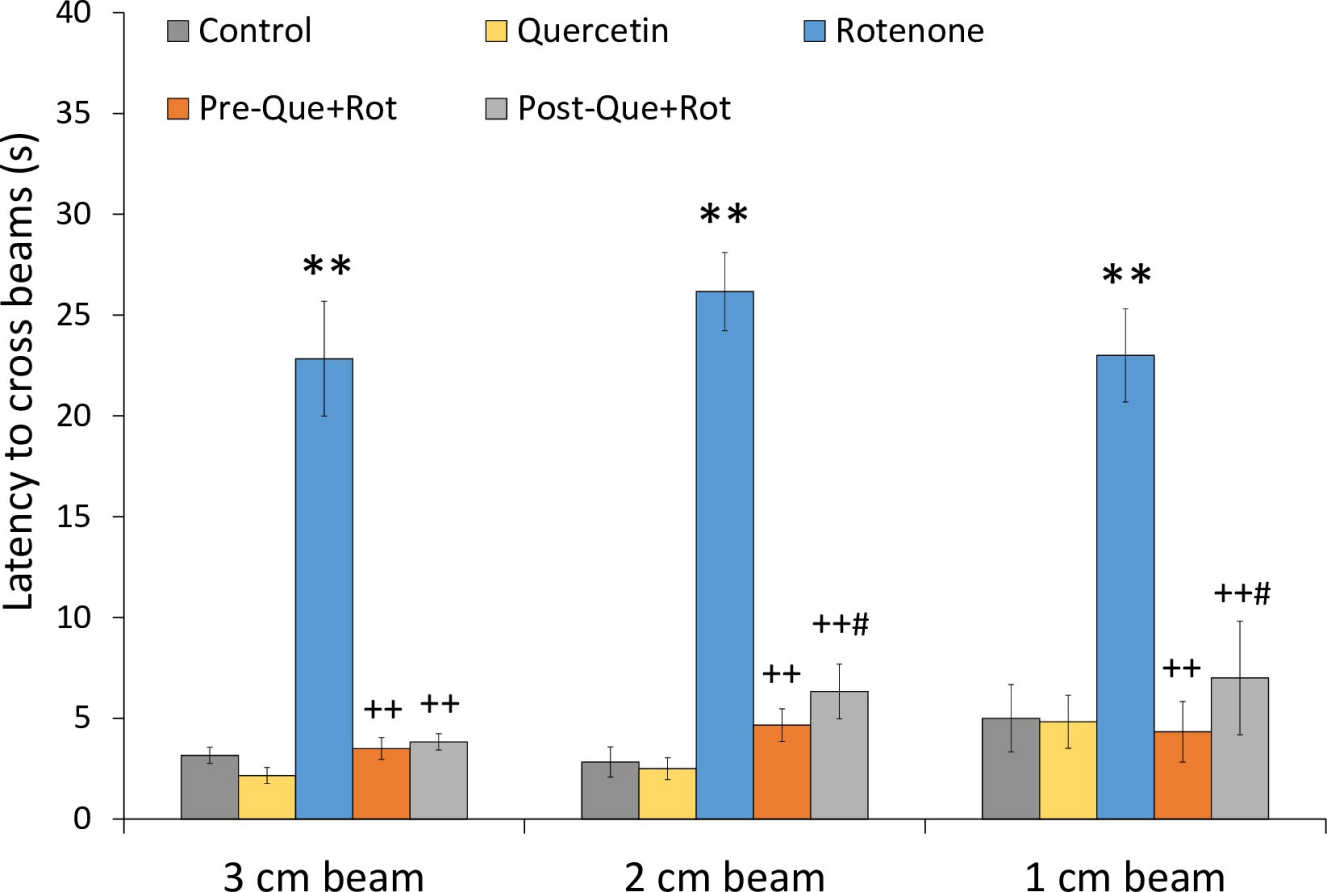
Effects of pre- and post-supplementation of quercetin on beam walking test in rotenone-injected rats. Significant differences were obtained by one-way ANOVA followed by Tukey’s test. **p<0.01 as compared to the control group; ++*p*<0.01 as compared to rotenone group; #*p*<0.05 as compared to Pre-Que+Rot group. Values are mean±SD (*n = 8*).

Beam walking test was used to monitor balance and motor coordination (Fig 5). This was accomplished by using beams with different diameters including 3, 2, and 1 cm. The data was analyzed using one-way ANOVA, which revealed that treatment had a significant influence on latency to cross 3 cm beam [*F* (4, 35) = 485.506, *p*<0.01], 2 cm beam [*F* (4, 35) = 780.653, *p*<0.01] as well as 1 cm beam [*F* (4, 35) = 180.032, *p*<0.01]. When compared to the control group, rotenone administration caused a substantial loss of motor control as demonstrated by significantly higher latency to cross (*p*<0.01) all sizes of beams. Whereas, the latency to cross all sizes of beams was considerably (*p*<0.01) decreased in both quercetin supplemented rats as compared to the rotenone group.

**Fig 6:**
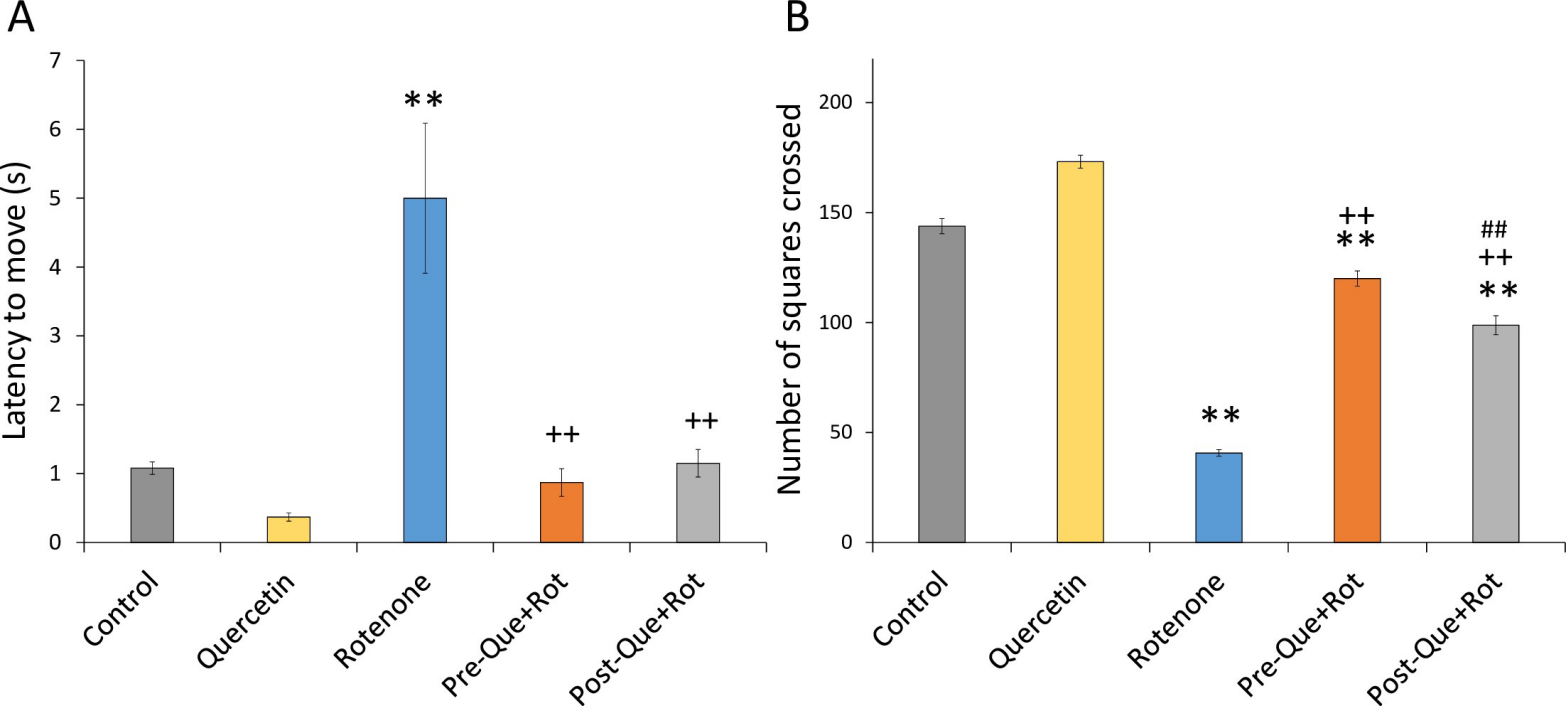
Effect of quercetin pre- and post-supplementation on exploratory activity by open field test in terms of (A) latency to move (s) and (B) number of square crossed. Values are represented as mean±SD (*n* = 8). **p<0.01 as compared to the control group; ++*p*<0.01 as compared to rotenone group; ##*p*<0.01 as compared to Pre-Que+Rot group.

The ambulatory and locomotor activities were monitored by the OFT paradigm (Fig 6A and 6B). Treatment induced significant effects on latency to move [*F* (4, 35) = 151.157, *p*<0.01] and number of squares crossed [*F* (4, 35) = 2624.072, *p*<0.01] in open filed. Rotenone-treated rats showed a considerably longer latency to move (*p*<0.01) and decreased number of squares crossed (*p*<0.01) than control rats. However, in comparison to the rotenone group, quercetin supplemented groups showed significantly reduced latency to move (*p*<0.01) and increased number of squares crossed (*p*<0.01) in OFT showing improved ambulatory and locomotor activity. Furthermore, pre-supplementation of quercetin had more improving effects (*p*<0.01) on ambulatory and locomotor activity than post-supplementation.

**Fig 7:**
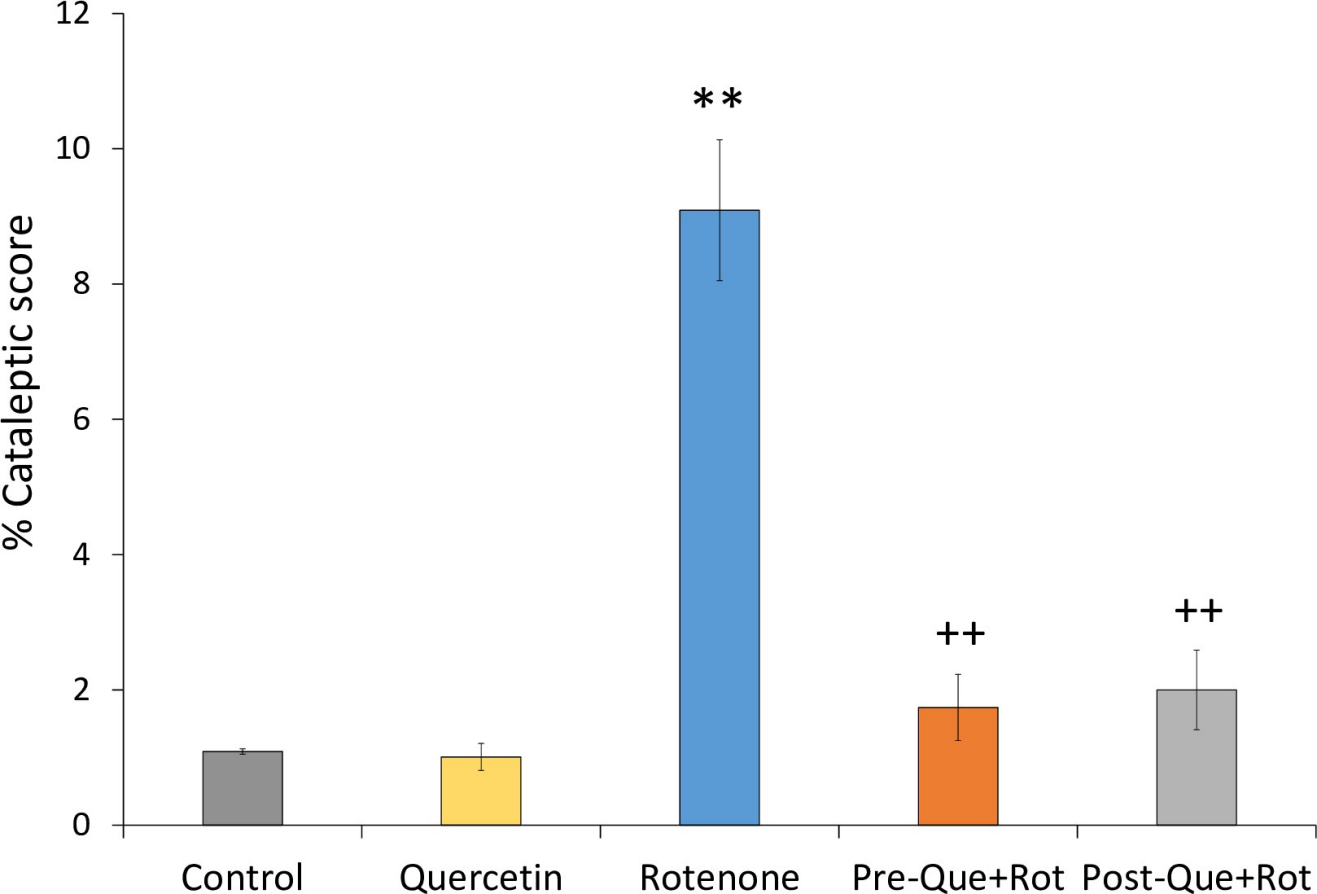
Effect of quercetin pre- and post-supplementation on rotenone-induced cataleptic condition in rotenone-injected rats. Values are represented as mean±SD (*n* = 8). Data was analyzed by Tukey’s test following one-way ANOVA. **p<0.01 as compared to the control group; ++*p*<0.01 as compared to rotenone group; ##*p*<0.01 as compared to Pre-Que+Rot group.

The inclined plane test was used to analyze cataleptic condition (Fig 7). One-way ANOVA revealed a significant effect of treatment on %cataleptic score [*F* (4, 35) = 375.756, *p*<0.01]. Post-hoc analysis showed a significant increase in %cataleptic score (*p*<0.01) in rotenone treated rats as compared to control animals. In comparison to rats injected with rotenone alone, quercetin supplementation before and after rotenone treatment resulted in a considerably lower %cataleptic score (*p*<0.01).

**Fig 8:**
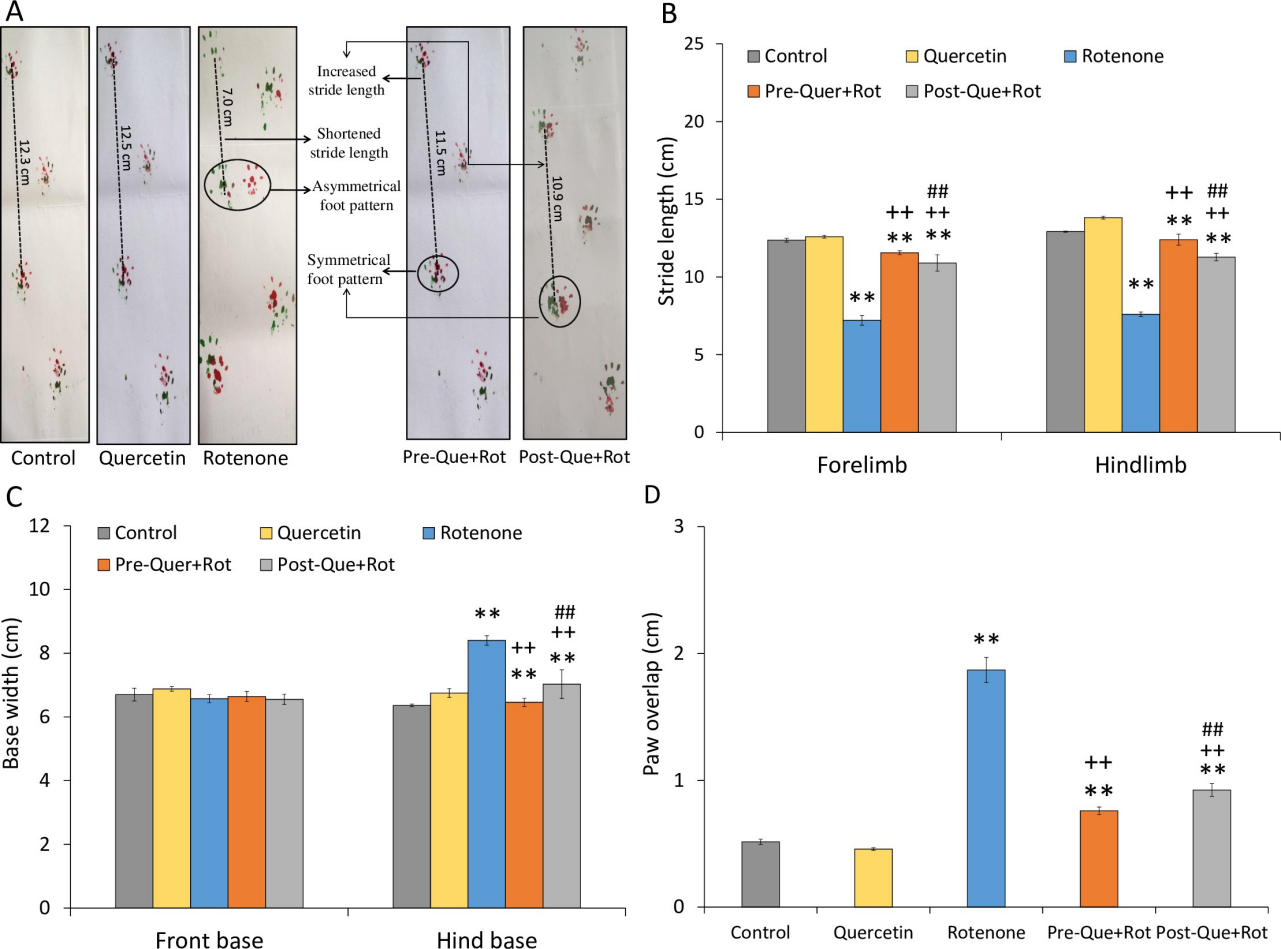
Walking pattern was attained by footprint test following pre-and post-supplementation of quercetin in rotenone-induced PD model (A) forepaws and hindpaws were coated with red and green ink. The dotted lines indicate stride length. The irregular distance between the fore- and hind paws in control and test rats is encircled. The quantification of (B) strides, (C) base width, and (D) paw overlap in all groups. **p<0.01 as compared to the control group; ++*p*<0.01 as compared to rotenone group; ##*p*<0.01 as compared to Pre-Que+Rot group.

The footprint of rats was also taken in this study to determine the walking pattern (Fig 8A–8D). The different parameters of walking pattern including forelimb stride length [*F* (4, 35) = 627.606, *p*<0.01], hindlimb stride length [*F* (4, 35) = 1419.317, *p*<0.01], front base width [*F* (4, 35) = 8.217, *p*<0.01], hind base width [*F* (4, 35) = 139.500, *p*<0.01], and overlapping of paws [*F* (4, 35) = 1158.189, *p*<0.01] were significantly affected by the given treatment. Tukey’s post-hoc test revealed that rotenone administration impaired walking pattern, as seen by substantially shorter (*p*<0.01) forelimb and hindlimb stride lengths as compared to control animals (Fig 8B). Moreover, there was a significant (*p*<0.01) increase in hind base width and paw overlapping, and a decrease in front base width due to rotenone administration as compared to controls. Quercetin, on the other hand, improved walking pattern in both pre- and post-supplementation groups. Both quercetin supplemented rats showed increased stride length of forelimb and hindlimb when compared with the rotenone group. In addition, as compared to rotenone group, hind base width and paw overlapping were considerably reduced in the Pre-Que+Rot and Post-Que+Rot groups. A more improved walking pattern was observed following pre-treatment with quercetin.

### Quercetin supplementation improved non-motor symptoms of PD

**Fig 9:**
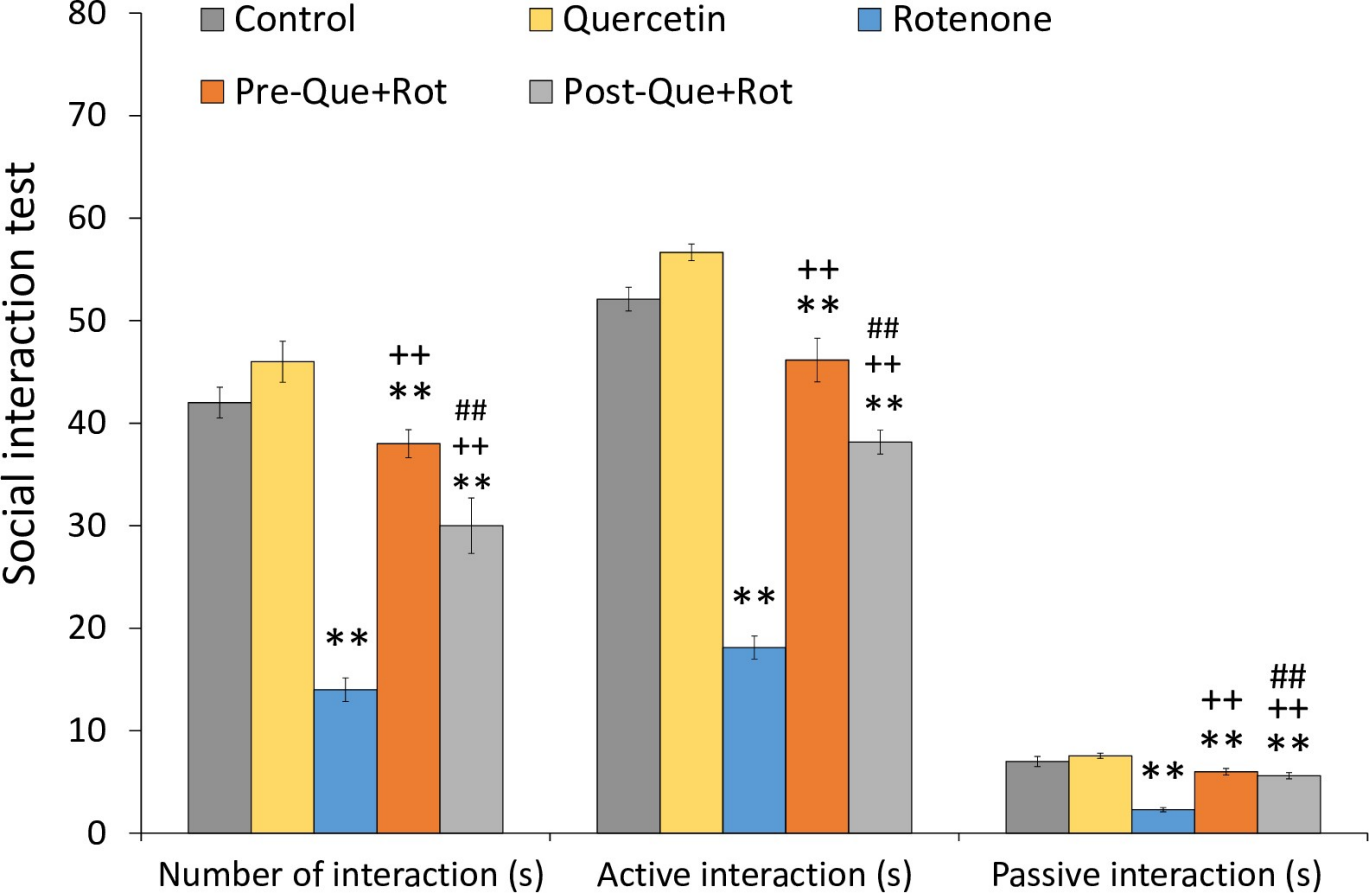
Depressive-like symptom following pre- and post-quercetin supplementation in rotenone-induced PD model were monitored by social interaction test. Values are represented as mean±SD (*n* = 8). Data was analyzed by Tukey’s test following one-way ANOVA. **p<0.01 as compared to the control group; ++*p*<0.01 as compared to rotenone group; ##*p*<0.01, #*p*<0.05 as compared to Pre-Que+Rot group.

The non-motor symptoms assessed in this study were depression-like behavior and cognitive function. Social interaction test was used to monitor depression-like behavior (Fig 9). One-way ANOVA revealed significant effects of treatment on number of interactions [*F* (4, 35) = 499.486, *p*<0.01], active interaction [*F* (4, 35) = 1395.077, *p*<0.01], and passive interaction [*F* (4, 35) = 433.897, *p*<0.01]. Tukey’s post-hoc analysis revealed that rotenone treatment considerably reduced the number of interactions in rats, as evidenced by a significant reduction in the number of interactions (*p*<0.01), active interaction (*p*<0.01), and passive interaction (*p*<0.01) as compared to control animals. However, pre- and post-supplementation of quercetin considerably increased the number of interactions (*p*<0.01), active interaction (*p*<0.01), and passive interaction (*p*<0.01) compared to rotenone-injected animals. Furthermore, pre-supplementation of quercetin had a higher significant influence on the number of interactions and active interaction (*p*<0.01) as compared to post-supplementation of quercetin.

**Fig 10:**
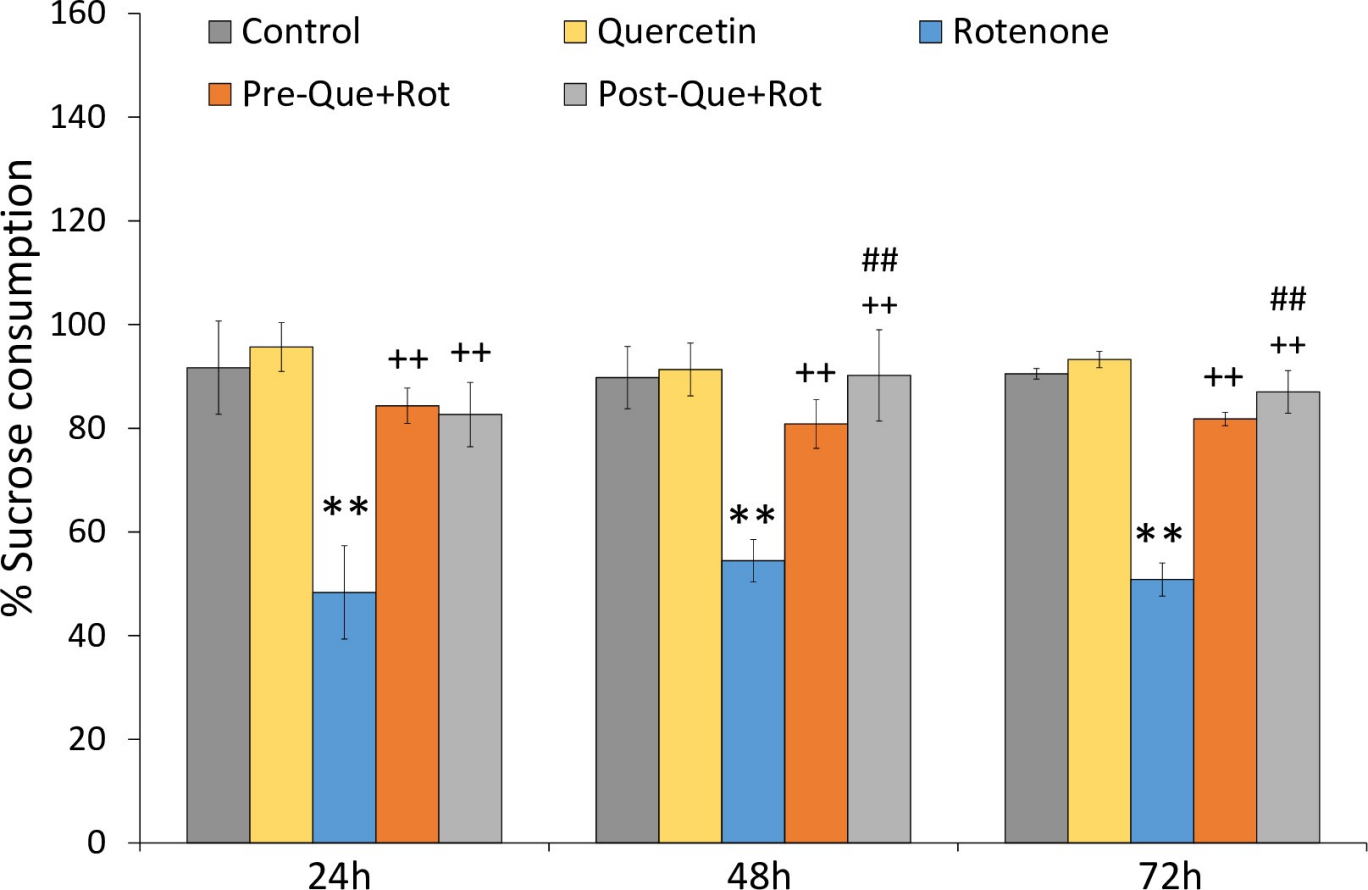
Depressive-like symptom following pre- and post-quercetin supplementation in rotenone-induced PD model were monitored by sucrose preference test. Values are represented as mean±SD (*n* = 8). Data was analyzed by Tukey’s test following one-way ANOVA. **p<0.01 as compared to the control group; ++*p*<0.01 as compared to rotenone group; ##*p*<0.01 as compared to Pre-Que+Rot group.

Anhedonia in rats was defined as the inability to experience pleasure from the intake of sugar. One-way ANOVA revealed that the treatment had a significant effect on sucrose solution intake measured after 24 h, [*F* (4, 35) = 82.641, *p*<0.01], 48 h [*F* (4, 35) = 70.931, *p*<0.01], and 72 h [*F* (4, 35) = 502.185, *p*<0.01]. The rats treated with rotenone took substantially (*p*<*0*.01) less sucrose solution than the controls. Whereas, quercetin supplemented groups took more sucrose solution after 24 h (*p*<0.01), 48 h (*p*<0.01), and 72 h (*p*<0.01) as compared to the group treated with rotenone alone (Fig 10).

**Fig 11:**
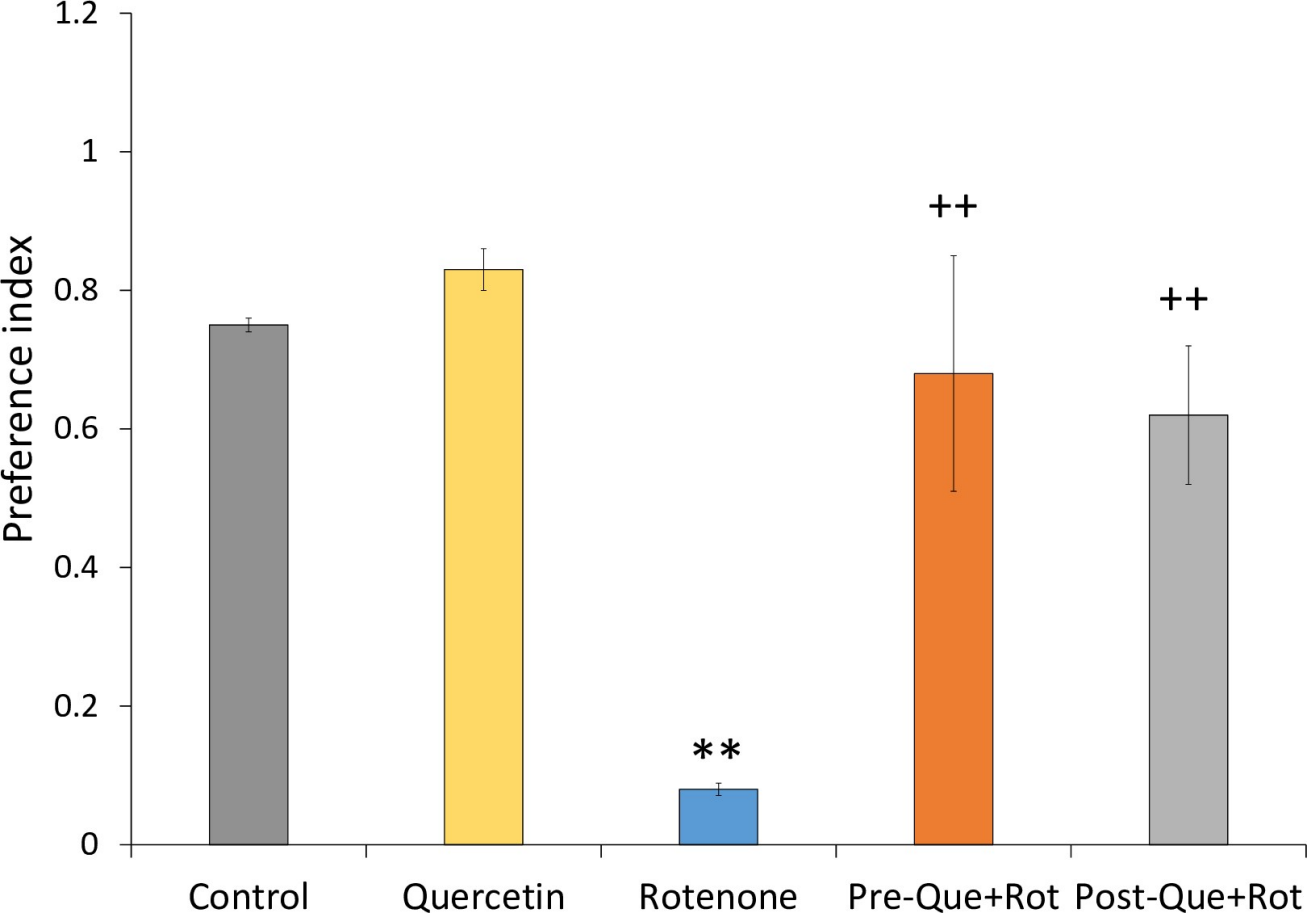
Effect of pre- and post-administration of quercetin (50 mg/kg) in rotenone-induced memory impairment was monitored by novel object recognition test. Values are mean±SD (*n = 8*). Data was analyzed by one-way ANOVA followed by Tukey’s post-hoc test. **p<0.01 as compared to the control group; ++*p*<0.01 as compared to rotenone group.

NOR task was used to assess cognitive function following quercetin and rotenone administration (Fig 11). Analysis of data by one-way ANOVA revealed a significant effect of treatment on preference index [*F* (4, 35) = 120.553, *p*<0.01]. Tukey’s post-hoc test showed that the group treated rotenone exhibited significantly (*p*<0.01) decreased preference as compared to the control animals. However, as compared to the rotenone group, quercetin supplementation had a substantial effect on rotenone-induced cognitive deficits, as evidenced by a significant increase in preference index (*p*<0.01).

**Fig 12:**
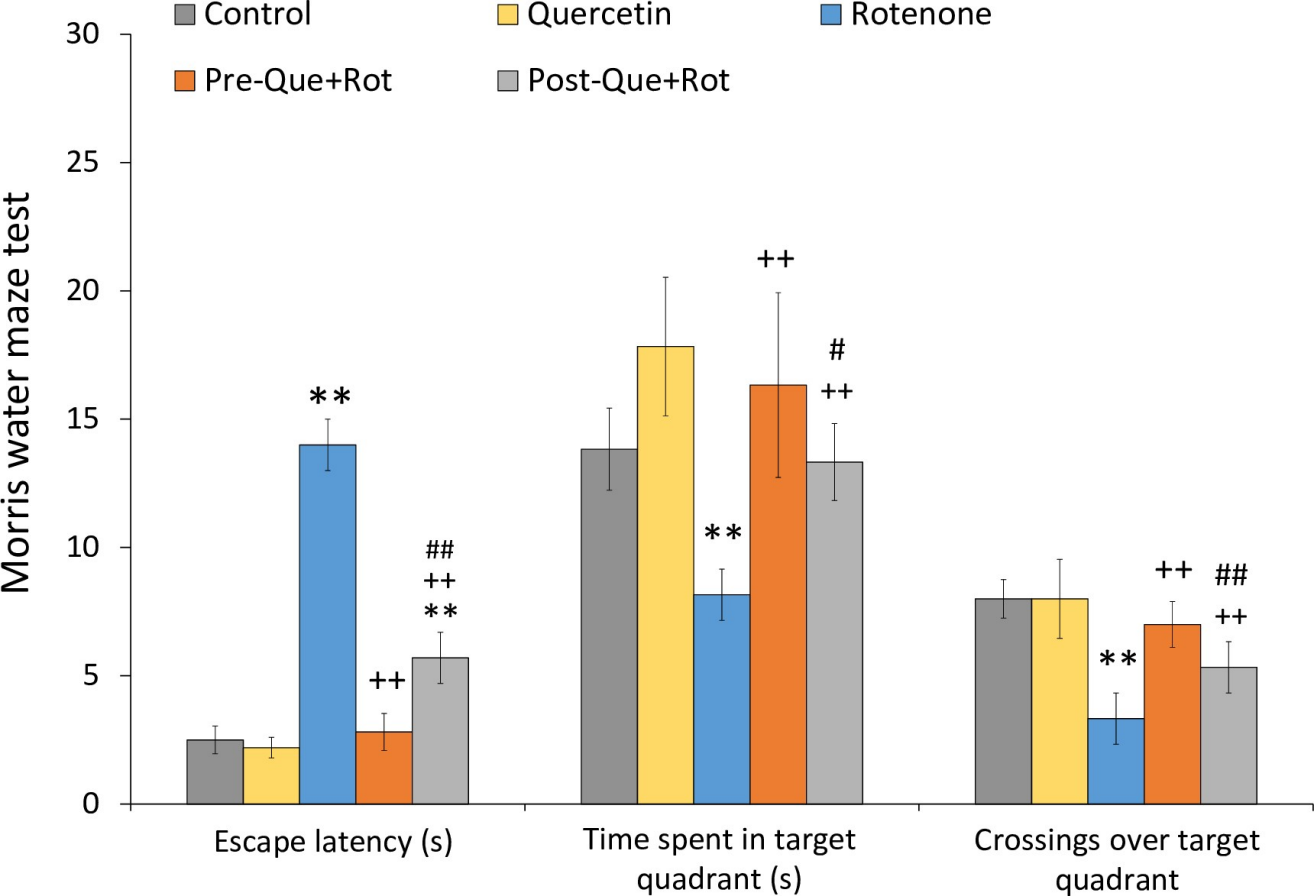
Effect of pre- and post-administration of quercetin in rotenone-induced memory impairment was monitored by Morris water maze test. Values are mean±SD (*n = 8*). Data was analyzed by one-way ANOVA followed by Tukey’s post-hoc test. **p<0.01 as compared to the control group; ++p<0.01 as compared to rotenone group; ##*p*<0.01, #*p*<0.05 as compared to Pre-Que+Rot group.

MWM test was employed to analyze the effects of treatment on memory function (Fig 12). Data analysis by one-way ANOVA showed a significant effect of treatment on escape latency [*F* (4, 35) = 180.999, *p*<0.01], time spent in the target quadrant [*F* (4, 35) = 28.689, *p*<0.01], and number of crossings over the target quadrant [*F* (4, 35) = 34.89, *p*<0.01]. Rotenone treatment exerted a significant increase in escape latency (*p*<0.01), and a significant decrease in time spent (*p*<0.01) and crossings over the target quadrant (*p*<0.05) as compared to the control animals. On the other hand, quercetin supplementation significantly reduced escape latency (*p*<0.01), and increased time spent (*p*<0.01) and crossings over the target quadrant (*p*<0.01) as compared to the rotenone group. Furthermore, quercetin pre-supplementation had more prominent effects than quercetin post-supplementation.

### Quercetin supplementation improved striatal and hippocampal biogenic amines

**Fig 13:**
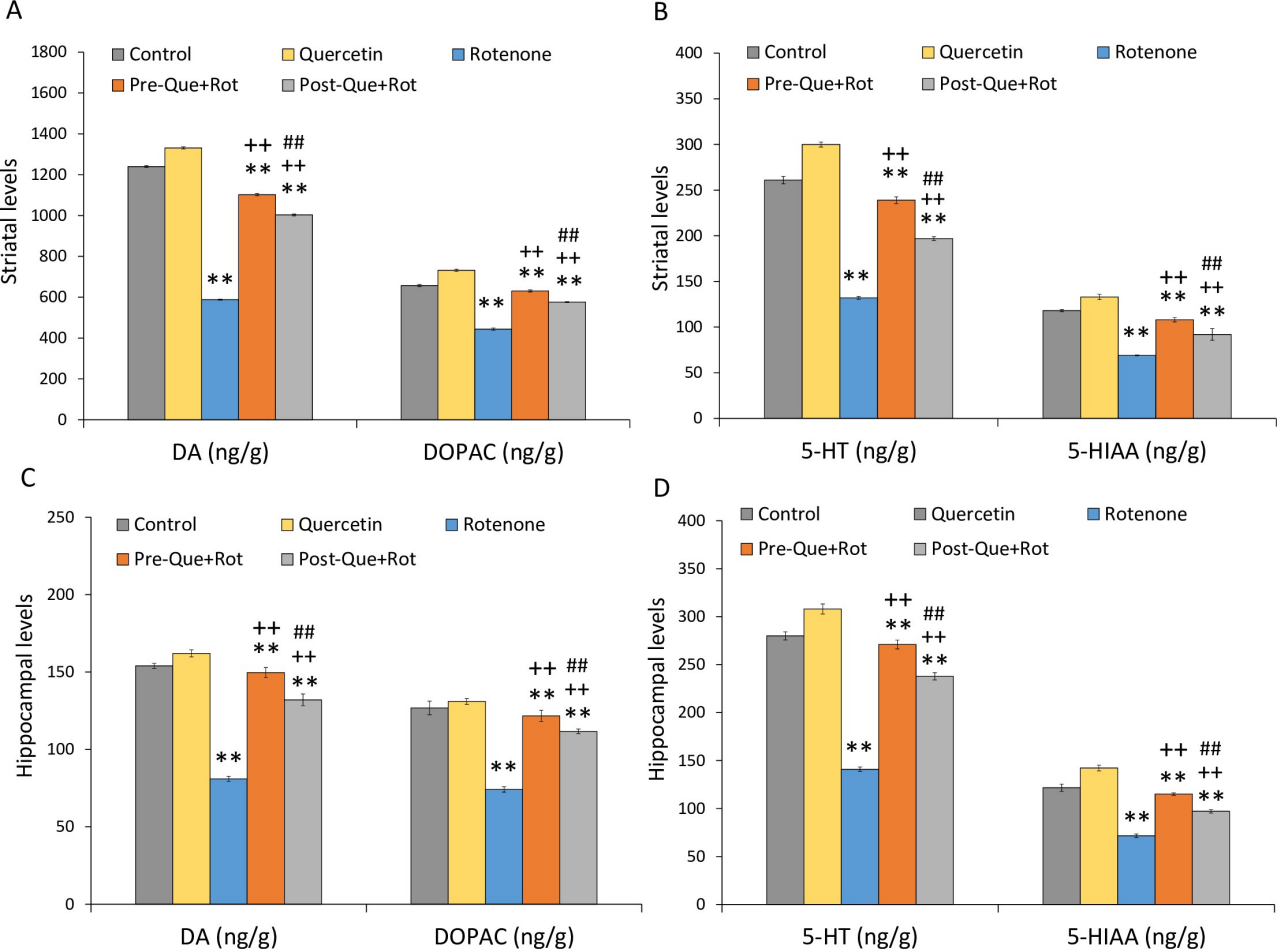
Neurotransmitter levels following pre- and post- supplementation of quercetin in rotenone-injected rats were estimated by HPLC in (A,B) striatum, (C,D) hippocampus. Values are mean±SD (*n = 8*). Significant differences were obtained by one-way ANOVA following Tukey’s test. **p<0.01 as compared to the control group; ++*p*<0.01 as compared to rotenone group; ##*p*<0.01 as compared to Pre-Que+Rot group.

Following the treatment of quercetin and rotenone, the change in biogenic amines in the striatum and hippocampus was evaluated using HPLC. There was a significant effect of treatment on DA [*F* (4, 35) = 47314.732, *p*<0.01], DOPAC [*F* (4, 35) = 5512.467, *p*<0.01], 5-HT [*F* (4, 35) = 4929.296, *p*<0.01], and 5-HIAA [*F* (4, 35) = 591.364, *p*<0.01] levels in the striatum (Fig 13A and 13B). When compared to controls, rotenone treatment resulted in a significant decrease in DA (*p*<0.01), DOPAC (*p*<0.01), 5-HT (*p*<0.01), and 5-HIAA (*p*<0.01) levels in the striatum. Pre-and post-supplementation of quercetin, however, normalized the loss of neurotransmitters in the striatum as shown by a considerable increase in DA (*p*<0.01), DOPAC (*p*<0.01), 5-HT (*p*<0.01), and 5-HIAA (*p*<0.01) levels as compared to rotenone injected rats. Furthermore, quercetin pre-supplementation had a more significant effect on neurotransmitter levels (*p*<0.01) than quercetin post-supplementation.

Analysis of biogenic amine levels was also carried out in hippocampus (Fig 13C and 13D). One-way ANOVA analysis showed a significant effect of treatment on DA [*F* (4, 35) = 1554.917, *p*<0.01], DOPAC [*F* (4, 35) = 704.885, *p*<0.01], 5-HT [*F* (4, 35) = 2642.922, *p*<0.01], and 5-HIAA [*F* (4, 35) = 1218.956, *p*<0.01] levels in hippocampus. When compared to the control group, the rats given rotenone showed a significant reduction in DA (*p*<0.01), DOPAC (*p*<0.01), 5-HT (*p*<0.01), and 5-HIAA (*p*<0.01) levels in the hippocampus. However, as compared to the rotenone alone group, quercetin supplementation substantially increased DA (*p*<0.01), DOPAC (*p*<0.01), 5-HT (*p*<0.01), and 5-HIAA (*p*<0.01).

### Quercetin supplementation improved striatal cholinergic function

**Fig 14:**
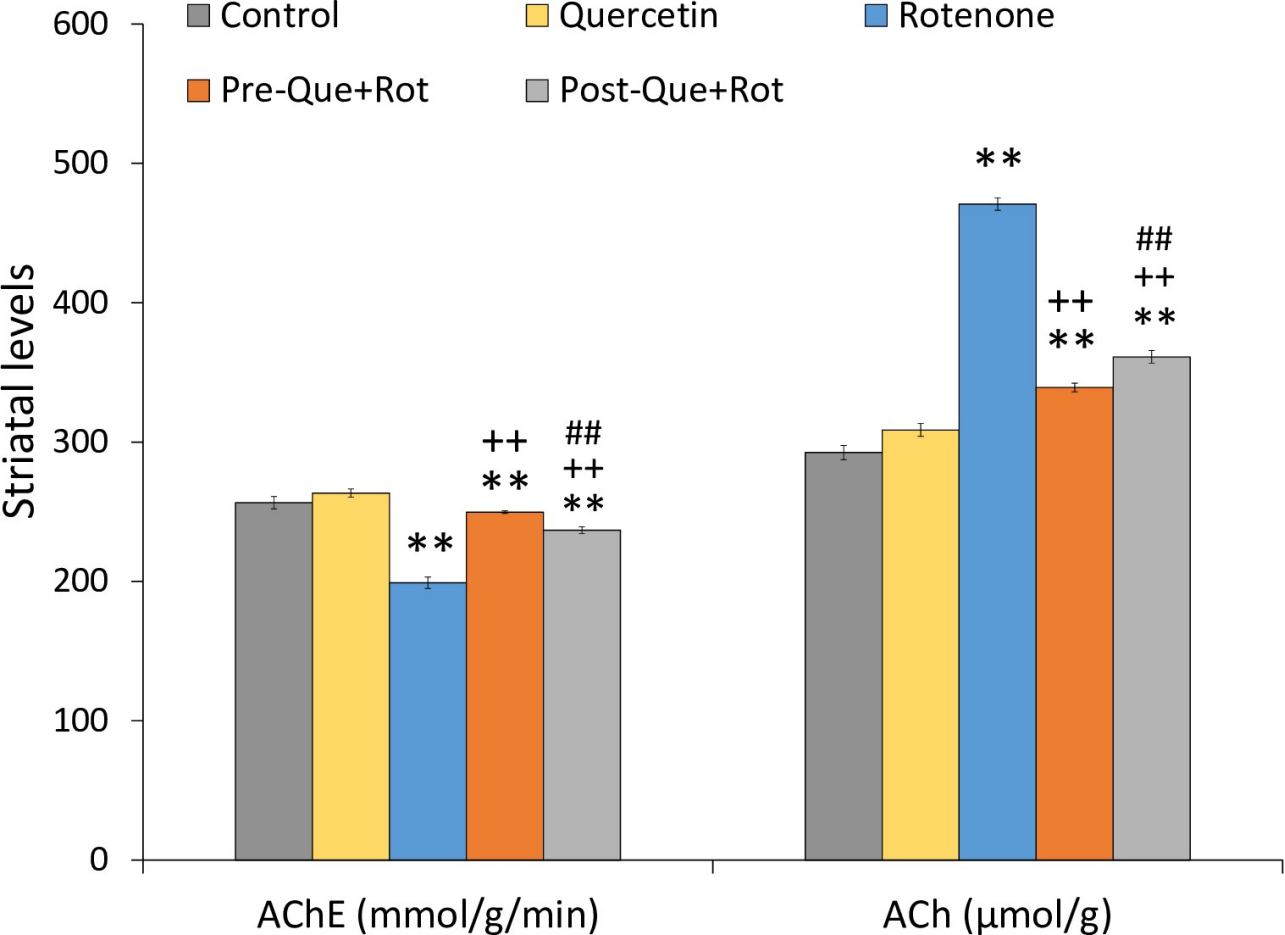
Effect of pre- and post-supplementation of quercetin on striatal acetylcholinesterase (mmol/g/min) activity, acetylcholine (μmol/g) levels in rotenone-injected rats. Values are mean±SD (*n* = 8) Significant differences were obtained by one-way ANOVA following Tukey’s test. **p<0.01 as compared to the control group; ++*p*<0.01 as compared to rotenone group; ##*p*<0.01 as compared to Pre-Que+Rot group.

Striatal cholinergic function was determined in terms of AChE activity and ACh levels (Fig 14). Data analysis by one-way ANOVA revealed a significant effect of treatment on AChE activity [*F* (4, 35) = 688.310, *p*<0.01] and ACh levels [*F* (4, 35) = 2817.696, *p*<0.01] in striatum. When compared to the control group, rotenone treatment substantially reduced (*p*<0.01) AChE activity and significantly raised (*p*<0.01) ACh levels. However, pre- and post-supplementation of quercetin considerably increased AChE activity (*p*<0.01) and significantly decreased ACh levels (*p*<0.01) in Pre-Que+Rot and Post-Que+Rot groups compared to the rotenone group moreover, pre-treatment with quercetin significantly (*p*<0.01) improved cholinergic functions as compared to the post-treatment group.

### Quercetin supplementation reduced rotenone-induced oxidative stress

**Fig 15:**
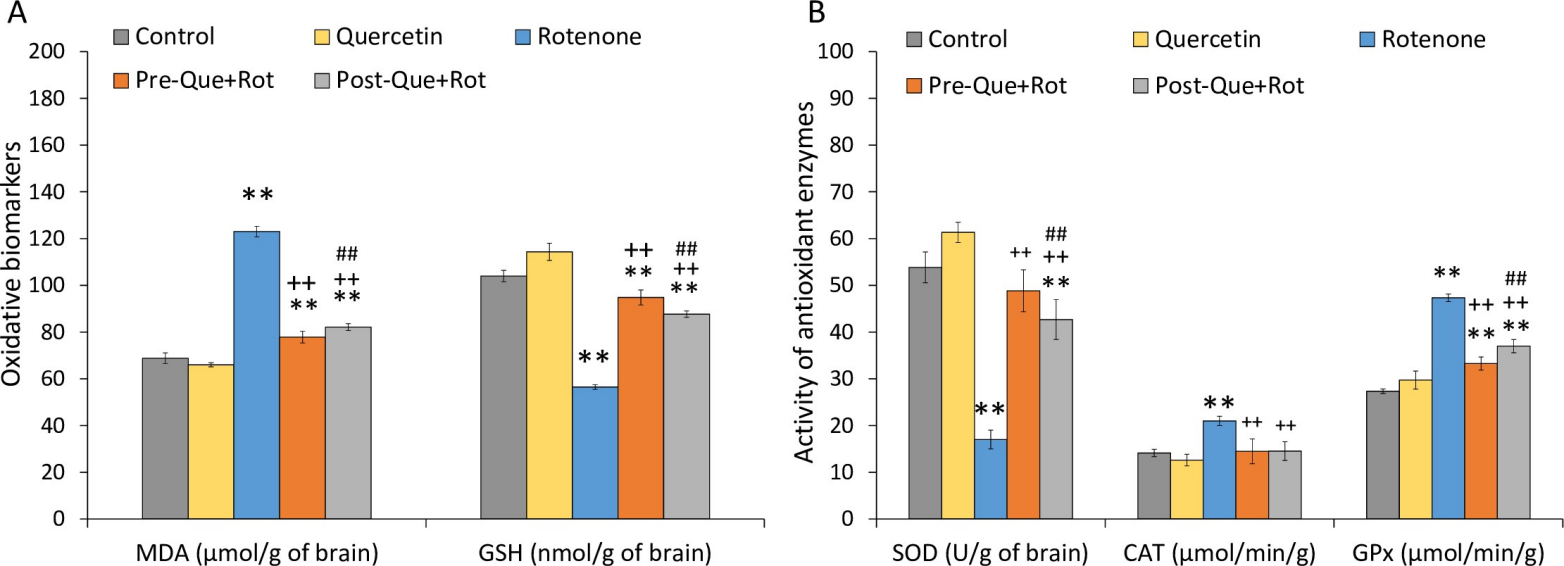
Effect of pre- and post-supplementation of quercetin on brain (A) malondialdehyde (μmol/g of brain tissue), reduced glutathione (nmol/g of brain tissue) levels and (B) antioxidant enzymes activity such as superoxide dismutase (U/g), catalase (μmol/min/g), and glutathione peroxidase (μmol/min/g) were also estimated. Values are mean±SD (*n* = 8). Significant differences were obtained by one-way ANOVA following Tukey’s test. **p<0.01 as compared to the control group; ++*p*<0.01 as compared to rotenone group; ##*p*<0.01 as compared to Pre-Que+Rot group.

Oxidative stress biomarkers including MDA and GSH levels were estimated in brain samples of rats. Data showed a significant effect of treatment on MDA [*F* (4, 35) = 1503.387, *p*<0.01] and GSH levels [*F* (4, 35) = 830.524, *p*<0.01] (Fig 15A). Tukey’s post-hoc test revealed that the rotenone-treated group exhibited a significant rise in MDA levels (*p*<0.01) and a significant reduction in GSH levels (*p*<0.01) as compared to controls. However, when compared to rotenone-treated rats, pre- and post-administration of quercetin significantly reduced MDA levels (*p*<0.01) and raised GSH levels (*p*<0.01). In comparison to the post-treatment, quercetin pre-treatment had more beneficial benefits (*p*<0.01) in reducing oxidative stress. The activity of antioxidant enzymes including SOD, CAT, and GPx was also determined (Fig 15B). Statistical analysis by one-way ANOVA showed a significant effect of treatment on SOD [*F* (4, 35) = 273.154, *p*<0.01], CAT [*F* (4, 35) = 42.065, *p*<0.01], and GPx [*F* (4, 35) = 396.283, *p*<0.01]. Post-hoc analysis revealed that the SOD was significantly reduced whereas CAT and GPx activity was significantly raised in rotenone injected rats when compared with the control animals (*p*<0.01). However, both quercetin supplemented groups exhibited significantly increased SOD activity, and reduced CAT and GPx activity as compared to rotenone group (*p*<0.01). However, pre-treatment of quercetin exhibited more enhancing effects on antioxidant levels as compared to the post-treatment of quercetin.

## Discussion

The present study evaluated the neuroprotective effects of quercetin against rotenone-induced neurotoxicity in rats. The major finding of this study was that both pre- and post-supplementation of quercetin significantly attenuated the motor and non-motor symptoms, and improved neurochemical and biochemical alterations induced by rotenone. The results showed that the rotenone-induced oxidative stress was significantly attenuated by the supplementation of quercetin. Extensive evidence shows that oral administration of quercetin in rodents provides protection from oxidative stress and exerts neuroprotective effects [26,56]. In the current study, supplementation of quercetin increased the SOD activity and decreased CAT and GPx activity. Moreover, quercetin supplementation increased GSH levels and decreased LPO. SOD is one of the main enzymes which convert O^-2^ into H_2_O_2_ and results in the normalization of CAT activity which catabolizes H_2_O_2_ into water. Quercetin a flavonol exerts its action by direct interaction with GPx and results in the modulation of GPx activity [56]. Our findings showed that quercetin supplementation normalized the GPx activity by increasing the GSH levels to protect against oxidative stress. It has been stated earlier that decreased GSH levels induced oxidative stress in the brain of PD patients [57]. Evidence showed that flavonoids protect the neuronal cells from neurodegeneration by increasing the activity of endogenous antioxidant enzymes [58]. Hence, it may be suggested that quercetin protects against rotenone-induced oxidative stress by increasing the activity of SOD and GSH levels which is consistent with the earlier findings using 6-OHDA and MPTP model of PD [20,59]. A substantial body of evidence suggests that oxidative damage produced by increased production of free radicals is associated with the alteration of endogenous antioxidant enzyme system [58]. It has been reported that flavonoids exert their action either by scavenging the free radicals or by directly increasing the activity of the antioxidant enzyme system [32,58]. Decreased LPO may be related to the increased activity of antioxidant enzymes following quercetin administration [60]. Hence, reduction in LPO by quercetin may help in delaying the neurodegenerative processes in PD.

Neurochemical analysis has shown decreased levels of DA, DOPAC, 5-HT, and 5-HIAA in the hippocampus and striatum following rotenone administration as mentioned earlier [40,61]. Degeneration of dopaminergic neurons in PD is caused by the mitochondrial dysfunction and increased production of ROS that affects the membrane integrity leading to the death of dopaminergic neurons [62,63]. Rotenone induces selective toxicity to DAnergic neurons by the inhibition of mitochondrial complex-I resulting in depletion of DA in the substantia nigra and nigrostriatal pathway [64]. We observed decreased levels of DA in the striatum following the administration of rotenone as reported previously [64]. Quercetin previously has been shown to improve the oxidative status in MPTP model of PD [59]. However, the protective effects of quercetin on rotenone-induced striatal and hippocampal neurotransmitter levels are not reported earlier. Here we are reporting that supplementation of quercetin improved biogenic amines and metabolites in the hippocampus and striatum of the rotenone rat model showing reduced neurodegeneration via the inhibition of oxidative stress. Vauzour et al. [65] reported that flavonoids stimulate neuronal regeneration and provide neuroprotection by strengthening neuronal function. Besides DAnergic degeneration, the intraneuronal aggregation and accumulation of α-synuclein also play a central role in the pathogenesis of PD. The *in vitro* study on PC12 cells has shown the aggregation of α-synuclein following the exposure of rotenone [66]. Quercetin has been described to inhibit α-synuclein aggregation [67]. Therefore, the neuroprotective role of quercetin in inhibiting the rotenone-induced aggregation of α-synuclein can also be suggested in this study. The role of DA and ACh is well reported in motor control. The interaction between DA and ACh is suggested to be responsible for motor and cognitive functions. Loss of DA neurons in PD results in the loss of control on ACh production and is considered to be the cause of dyskinesia in PD patients [68]. In the striatum, cholinergic interneurons play an important role in the pathophysiology of PD. The actions of DA on D_2_ receptors present on striatal cholinergic interneurons cause a decrease in striatal ACh release. Therefore, in PD reduced striatal DA leads to overstimulation of cholinergic interneurons resulting in excess release of ACh in the striatum as observed in the present study [69,70]. AChE is an enzyme that degrades the ACh, which is an excitatory neurotransmitter. Our findings showed that rotenone administration significantly decreased the AChE activity resulting in increased levels of ACh in the striatum. Inhibition of AChE activity is suggested to aggravate the motor impairment of PD. Anticholinergic drugs, therefore, have a role in reducing the symptoms of PD, particularly the associated tremors [71]. Quercetin supplementation, however, attenuated the rotenone-induced reduction in AChE activity suggesting that quercetin supplementation reduced the ACh levels by increasing AChE activity, which is in line with the previous study [57]. Hence, present findings suggest that restoration of AChE activity by quercetin may be attributed to the relief of many of the locomotor impairments induced by rotenone. Administration of rotenone induced PD-like motor deficits in rats while quercetin markedly improved motor control, balance, and coordination in this study. An earlier study has shown that quercetin treatment plays an important role in the development of internal processes and regulates motor behavior [59]. The improved motor functions following quercetin supplementation may be attributed to the increased striatal DA levels.

Like motor symptoms, non-motor symptoms in PD also have significance due to their presence before the disease diagnosis or appearance with the disease progression [72]. Results obtained from the present study show that rotenone administration significantly induced depressive-like symptoms and anhedonia as observed previously [40]. Dopaminergic and non-dopaminergic neurons (5-HT) play a significant role in the development of depression in PD [73]. In line with the previous studies, the present study showed that rotenone administration significantly reduced DA, 5-HT, and metabolite levels in the hippocampus and striatum which resulted in depressive-like symptoms in rats [10,74]. Quercetin supplementation in rotenone-injected rats produced antidepressant-like effects in the social interaction test and sucrose preference test. Quercetin treatment attenuated depressive behavior, increased sociability, and decreased anhedonia in rats, moreover, it also increased DA, 5-HT, and their metabolite levels. These results indicate that antidepressant ability of quercetin may be mediated by increased levels of DA and 5-HT. A previous study has shown the antidepressant effect of quercetin in streptozocin-induced diabetic mice [75]. The improvement in depressive-like symptoms and increased 5-HT levels may be attributed to the inhibition of MAO-A enzyme by quercetin as reported earlier [76,77].

**Fig 16:**
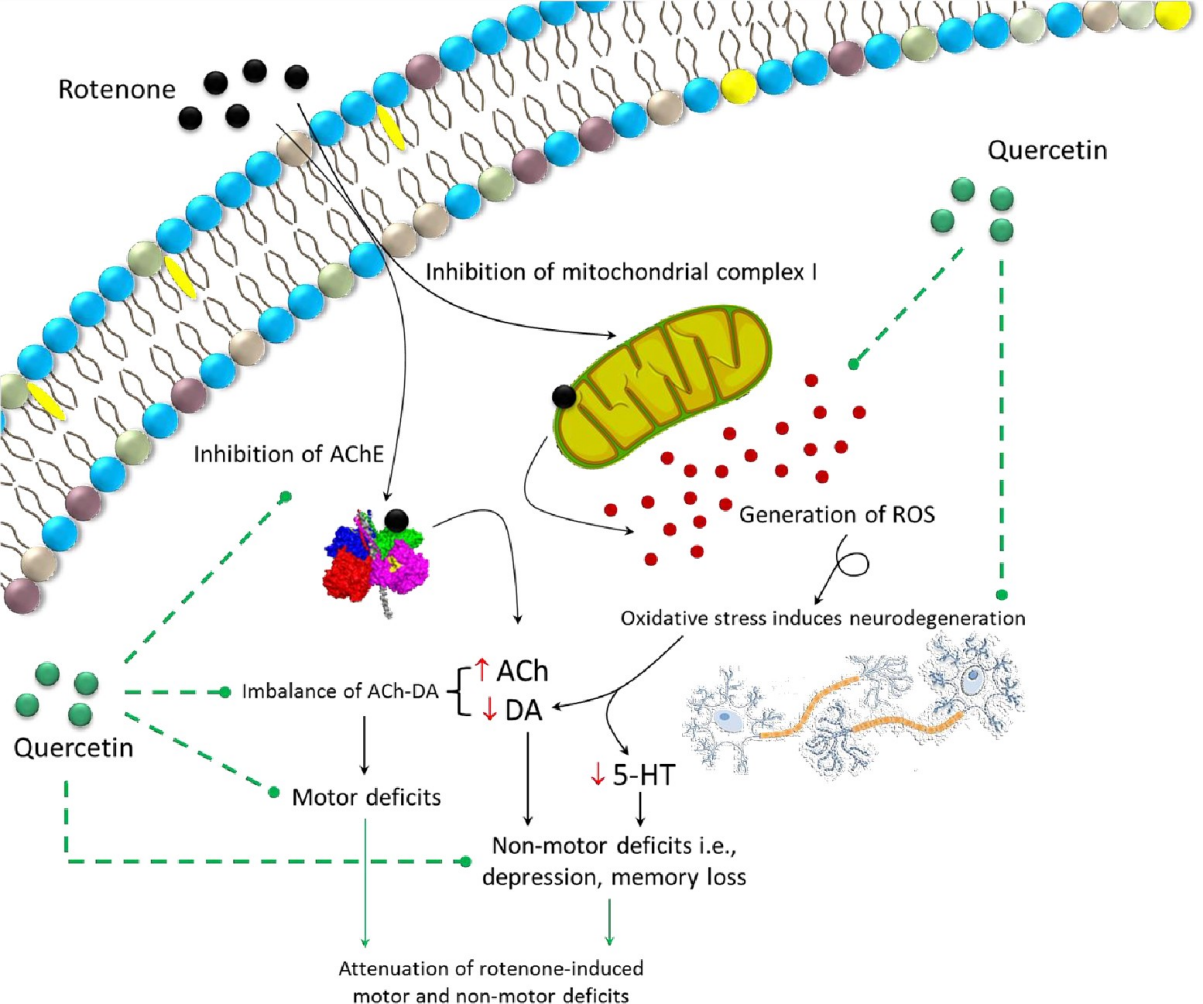
Summarized experimental findings showing inhibition of mitochondrial complex-I by rotenone causing the generation of reactive species and neuronal disruption. Rotenone is also found to inhibit the activity of acetylcholinesterase (AChE) enzyme. Oxidative stress and reduced activity of AChE lead to imbalance DA-ACh and reduced serotonin levels resulting in motor and non-motor PD-like symptoms. Availability of quercetin, on the other hand, reduces the oxidative stress, and regulates AChE activity and thus helps in attenuation of rotenone-induced motor and non-motor symptoms of PD.

Cognitive impairments in PD are evident at an early stage of the disease [78]. Rotenone administration in this study induced cognitive impairment which was assessed by MWM and NOR task. Quercetin supplementation before and after rotenone administration significantly improved memory performance as evident from decreased escape latency, increased time spent in target quadrant, and number of crossing over the target quadrant. The memory improving effects of quercetin were further confirmed by an increase in preference index in NOR. Studies from an animal model of PD have shown that decreased levels of DA and 5-HT following 6-OHDA and MPTP resulted in cognitive impairments [79,80]. Moreover, quercetin has also shown to improve memory function and attenuate neuronal death in the hippocampus [81]. The role of 5-HT and DA in cognitive functions is well documented [82,83]. Results indicated that decreased levels of DA and 5-HT by rotenone administration were significantly increased by quercetin treatment which improved memory function observed in MWM and NOR. The protective mechanism of quercetin might be due to a direct or indirect effect of quercetin by scavenging free radicals. The pre-supplementation of quercetin resulted in more prominent effects than post-supplementation. It has been suggested that the regular intake of flavonoid-containing food or beverages can reduce the risk of neurodegenerative diseases with advancing age [84]. Dietary consumption of fruit and vegetable juices rich in flavonoids has been strongly associated with the prevention of cognitive impairment with advancing age, delay in the onset of Alzheimer’s disease, and fewer chances of developing PD [85–88]. This study also provides scientific evidence for the preventive potential of quercetin against the development of PD, and thus can be recommended for individuals who are more at risk. Taken together, our study suggests that quercetin supplementation may improve neurotransmitter levels by reducing oxidative stress via increasing antioxidant enzyme activity and hence improves motor activity, cognitive functions, and reduces depression-like behavior (Fig 16). The histological examination confirms the neuronal loss associated with PD-like symptoms in animal model. This study does not include any such evidence, which is a major limitation. Therefore, in future, histological studies may provide more detailed evidence for rotenone-induced neuronal loss and its protection by quercetin supplementation.

## Conclusion

In conclusion, quercetin supplementation ameliorates the rotenone-induced motor, non-motor deficits, oxidative stress, and neurotransmitter alterations. The present findings suggest that both preventive and therapeutic regimens of quercetin can prevent rotenone-induced decreased levels of dopamine and serotonin through its potent antioxidant ability. However, quercetin pre-supplementation produces more significant results as compared to post-supplementation. Thus, it is suggested that quercetin can be used as a potential compound to reduce the risk and progression of PD.
